# The second window ICG technique demonstrates a broad plateau period for near infrared fluorescence tumor contrast in glioblastoma

**DOI:** 10.1371/journal.pone.0182034

**Published:** 2017-07-24

**Authors:** Ryan Zeh, Saad Sheikh, Leilei Xia, John Pierce, Andrew Newton, Jarrod Predina, Steve Cho, MacLean Nasrallah, Sunil Singhal, Jay Dorsey, John Y. K. Lee

**Affiliations:** 1 Department of Neurosurgery, Hospital of the University of Pennsylvania, Philadelphia, Pennsylvania, United States of America; 2 Center for Precision Surgery, Hospital of the University of Pennsylvania, Philadelphia, Pennsylvania, United States of America; 3 Department of Radiation Oncology, Hospital of the University of Pennsylvania, Philadelphia, Pennsylvania, United States of America; 4 Department of Surgery, Hospital of the University of Pennsylvania, Philadelphia, Pennsylvania, United States of America; 5 Department of Pathology, Hospital of the University of Pennsylvania, Philadelphia, Pennsylvania, United States of America; Stanford University, UNITED STATES

## Abstract

**Introduction:**

Fluorescence-guided surgery has emerged as a powerful tool to detect, localize and resect tumors in the operative setting. Our laboratory has pioneered a novel way to administer an FDA-approved near-infrared (NIR) contrast agent to help surgeons with this task. This technique, coined Second Window ICG, exploits the natural permeability of tumor vasculature and its poor clearance to deliver high doses of indocyanine green (ICG) to tumors. This technique differs substantially from established ICG video angiography techniques that visualize ICG within minutes of injection. We hypothesized that Second Window ICG can provide NIR optical contrast with good signal characteristics in intracranial brain tumors over a longer period of time than previously appreciated with ICG video angiography alone. We tested this hypothesis in an intracranial mouse glioblastoma model, and corroborated this in a human clinical trial.

**Methods:**

Intracranial tumors were established in 20 mice using the U251-Luc-GFP cell line. Successful grafts were confirmed with bioluminescence. Intravenous tail vein injections of 5.0 mg/kg (high dose) or 2.5 mg/kg (low dose) ICG were performed. The Perkin Elmer IVIS Spectrum (closed field) was used to visualize NIR fluorescence signal at seven delayed time points following ICG injection. NIR signals were quantified using LivingImage software. Based on the success of our results, human subjects were recruited to a clinical trial and intravenously injected with high dose 5.0 mg/kg. Imaging was performed with the VisionSense Iridium (open field) during surgery one day after ICG injection.

**Results:**

In the murine model, the NIR signal-to-background ratio (SBR) in gliomas peaks at one hour after infusion, then plateaus and remains strong and stable for at least 48 hours. Higher dose 5.0 mg/kg improves NIR signal as compared to lower dose at 2.5 mg/kg (SBR = 3.5 vs. 2.8; P = 0.0624). Although early (≤ 6 hrs) visualization of the Second Window ICG accumulation in gliomas is stronger than late (≥24 hrs) visualization (SBR = 3.94 vs. 2.32; p<0.05) there appears to be a long plateau period of stable ICG NIR signal accumulation within tumors in the murine model. We call this long plateau period the “Second Window” of ICG. In glioblastoma patients, the delayed visualization of intratumoral NIR signal was strong (SBR 7.50 ± 0.74), without any significant difference within the 19 to 30 hour visualization window (R^2^ = 0.019).

**Conclusion:**

The Second Window ICG technique allows neurosurgeons to deliver NIR optical contrast agent to human glioblastoma patients, thus providing real-time tumor identification in the operating room. This nonspecific tumor accumulation of ICG within the tumor provides strong signal to background contrast, and is not significantly time dependent between 6 hours to 48 hours, providing a broad plateau for stable visualization. This finding suggests that optimal imaging of the “Second Window of ICG” may be within this plateau period, thus providing signal uniformity across subjects.

## Introduction

Every year in the United States, there are approximately 23,000 new brain and nervous system cancer diagnoses [1]. Glioblastoma multiforme (GBM) is the most common malignant brain tumor in adults, accounting for about 45% of these cases. The one- and two-year survival rates of GBM are only 53% and 15%, respectively [2, 3]. Furthermore, median survival is only 15 months following standard treatment, which consists of maximal safe resection, followed by adjuvant radiation and chemotherapy. Surgical resection of GBM is an important first step as it provides relief of mass effect, allows for histopathologic diagnosis, and improves survival.

Intraoperative fluorescence imaging is an exciting technique that aids surgeons in the detection and resection of tumors in the operative setting [4]. Although 5-ALA has shown success in human glioblastoma trials, this visible light prodrug is not FDA-approved in the United States [5]. The only FDA-approved NIR contrast agent is indocyanine green (ICG). In the NIR range, ICG fluoresces with a peak emission and excitation at 780 and 810 nm respectively [6, 7]. Since initial FDA approval in 1958, there has been extensive research to improve the utility of ICG in the medical field. ICG is traditionally administered as a video angiographic contrast agent with doses between 0.2 mg/kg and 0.5 mg/kg. Since the serum half-life is measured only in minutes, this allows the surgeon to perform bolus injections for intraoperative vascular identification [8].

Our laboratory has successfully used ICG in a completely different manner from video-angiography. Specifically, we have pioneered a technique that we call Second Window ICG [9]. This technique requires delivery of doses as high as 5.0 mg/kg, and instead of imaging immediately following the infusion, the tumor is imaged approximately 24 hours later. This allows the dye to circulate throughout the body, and to accumulate in pathologic tumor tissue via the Enhanced Permeability and Retention (EPR) effect [10]. By exploiting this delivery method, NIR cameras can be used in the operating room environment to visualize ICG accumulation within tumors. Our lab has used this technique in a variety of tumor types, including lung, ovarian, colorectal, breast, and metastases [9, 11–17].

Previous pre-clinical experiments have studied dose and optimal timing for intravenous ICG delivery for tumor imaging. However, these studies were conducted with non-small cell lung cancer (NSCLC), mesothelioma, and esophageal carcinoma cell lines amongst others, with subcutaneous tumor models in mice [9]. Given that the brain is unique in having a relatively impermeable blood-brain barrier and is isolated from organs that are involved in the excretion of ICG (e.g. stomach, bowel, liver), we hypothesized that Second Window ICG can be used to visualize intracranial brain tumors. We subsequently tested different dosing and timing of visualization, as well as signal strength, in an intracranial tumor model, followed by careful analysis of human patients.

## Materials and methods

### Reagents

Pharmaceutical grade indocyanine green (ICG) was purchased from Akorn Inc. (IC-Green, NDC 17478-791-02). Animals were dosed with 2.5 or 5.0 mg/kg of ICG in 100 μL of deionized water via intravenous tail vein injection.

### Cell line

The U251-Luc-GFP malignant glioblastoma-astrocytoma cell line was obtained from collaborators at the University of Pennsylvania [18]. The cell line was cultured and maintained in DMEM media supplemented with 10% fetal bovine serum and 1% penicillin/streptomycin.

### Intracranial tumor model

Six-week-old female nude athymic mice (strain code 490) were purchased from Charles River Laboratories. All mice were maintained in pathogen-free conditions under University of Pennsylvania approved protocols.

Orthotopic models of brain tumors were created as described previously [18]. U251 cells were harvested, re-suspended in 10 uL PBS when they reached 70% confluency. Mice were pre-medicated with subcutaneous injection of 5.0 mg/kg of meloxicam in saline to control for post-operative pain and inflammation. Mice were then anesthetized with 2–4% isofluorane. The mice were positioned in a stereotactic frame. The injection site was then located 2.0 mm posterior and 1.5 mm lateral to the bregma in the right cerebral hemisphere. A small hole was drilled, and a stereotactically guided 30 gauge needle was used to sterilely deliver 3x10^5^ cells in 6 uL DMEM at a rate of 0.5 μL/min 3 mm deep into the cortex. The needle was left in the brain for two minutes following injection to prevent reflux. The hole was closed with bone wax and incision sealed with veterinary tissue glue [18].

### Bioluminescent imaging

The U251 cells were genetically modified to express firefly luciferase. This feature enables bioluminescent imaging (BLI) of living tumor cells after an injection of d-luciferin. Mice implanted with brain tumors were serially monitored with BLI every week until the tumor was determined to be of sufficient size. Anesthetized mice (2% isofluorane in 100% oxygen) were injected subcutaneously with 100 uL of a 50 mg/mL solution of D-Luciferin (GoldBio Inc.) in PBS. Imaging was performed using the IVIS (Perkin Elmer).

### NIR imaging and measurement of fluorescence intensity in animal model

When the intracranial tumor reached sufficient size, the mice were injected with ICG via tail vein and imaged at different time points (immediately following injection, 1 hour, 3 hours, 6 hours, 12 hours, 24 hours, and 48 hours post injection). NIR signal was captured using the PerkinElmer IVIS Spectrum machine (745 nm/840 nm). The IVIS Spectrum system is a closed field imaging system; the mice are placed in a dark enclosed chamber that minimizes extraneous ambient light.

**Fig 1:**
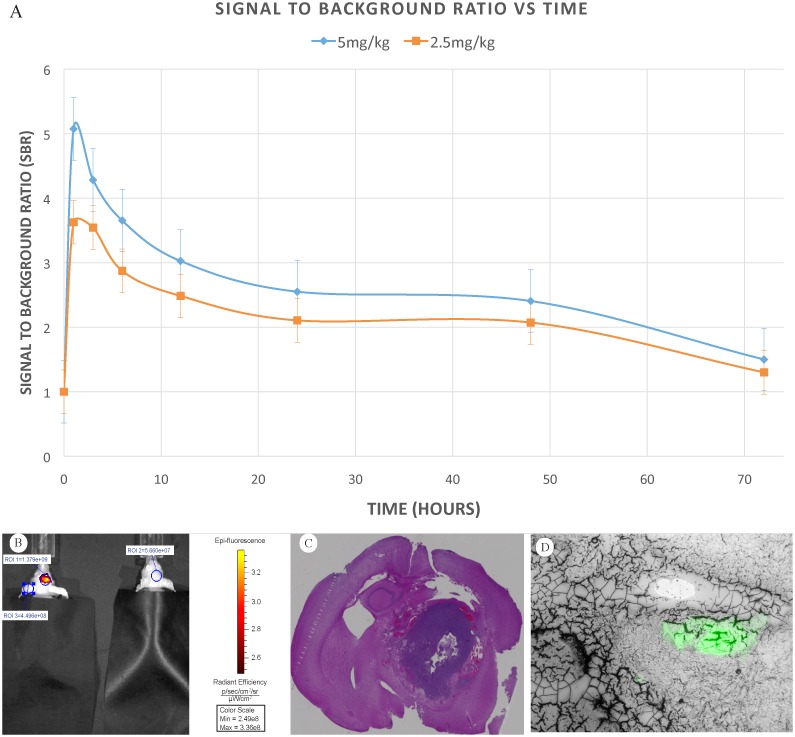
Second Window ICG in murine model. A) Signal to background ratio for mice in both 5.0mg/kg group and 2.5mg/kg group plotted versus time of imaging after ICG tail vein infusion. Point estimates at each time point represent average SBRs of the 10 mice in each group. Standard error bars are included. B) Illustration of mouse brain tumor 3 hours after administration of ICG (left) compared to control (no ICG infusion) mouse (right). Regions of interests used for quantifying fluorescence using LivingImage software are also shown. C) Coronal slice of animal’s entire brain stained with H&E. D) Epifluorescence microscopy of NIR signal in established tumor within mouse model.

After capturing the data, signal to background ratios (SBR) were generated using LivingImage software (PerkinElmer) in order to quantify the strength and specificity of the NIR signal from the IVIS. SBRs were obtained by comparing regions of interest (ROI) in the mice. The ROI for the ‘signal’ was placed at the tumor, just above the injection site, whereas the ROI for the ‘background’ was placed on adjacent tissue closer to the ear or nape of the neck (Fig 1). The LivingImage software then reports the fluorescent intensity from these regions in units of radiant efficiency (emission/excitation). Upon quantifying fluorescence with the LivingImage software, both total radiant efficiency (TRE) (photons s^-1^cm^-2^ steradian^-1^ / uW cm^-2^) and average radiant efficiency (ARE) (photons/s/cm^2^/steradian) / (uW/cm^2^) were analyzed **(**Fig 1). Average Radiant Efficiency units are presented in this analysis.

Of note, there was a difference in how the ROIs were placed in the mice versus the humans. In the mice the ROI was located at the center of the brightest signal intensity, which corresponded to the injection site of the tumor (Fig 1). The background ROI in mice, however, was chosen closer to the ear or nape of the neck. Similar to the mice, in the human study, the ROI was placed directly over the tumor. However, the choice of background in humans differed. In the humans, the background ROI was placed in adjacent brain parenchyma, because there was no overlying skull or skin in the human subjects. This difference in technique may account for the differences in SBR between murine and human subjects.

### Histological analysis

After serially imaging the mice and their tumors with the system, the mice were sacrificed and their brains were harvested. Brains were prepared for coronal sectioning, placed on slides and stained with hematoxylin and eosin for imaging on a color microscope (Fig 1C).

### Fluorescence microscopy

Animal brains were harvested after the final imaging time point. The brains were immediately placed into a prepared block of OTC Compound (Tissue-Tek) on dry ice. The block was then cut in 10 μm sections in coronal fashion. Slides were immediately placed under the microscope for examination. (Fig 1D)

### Human clinical trial

#### Study design and protocol

**Fig 2:**
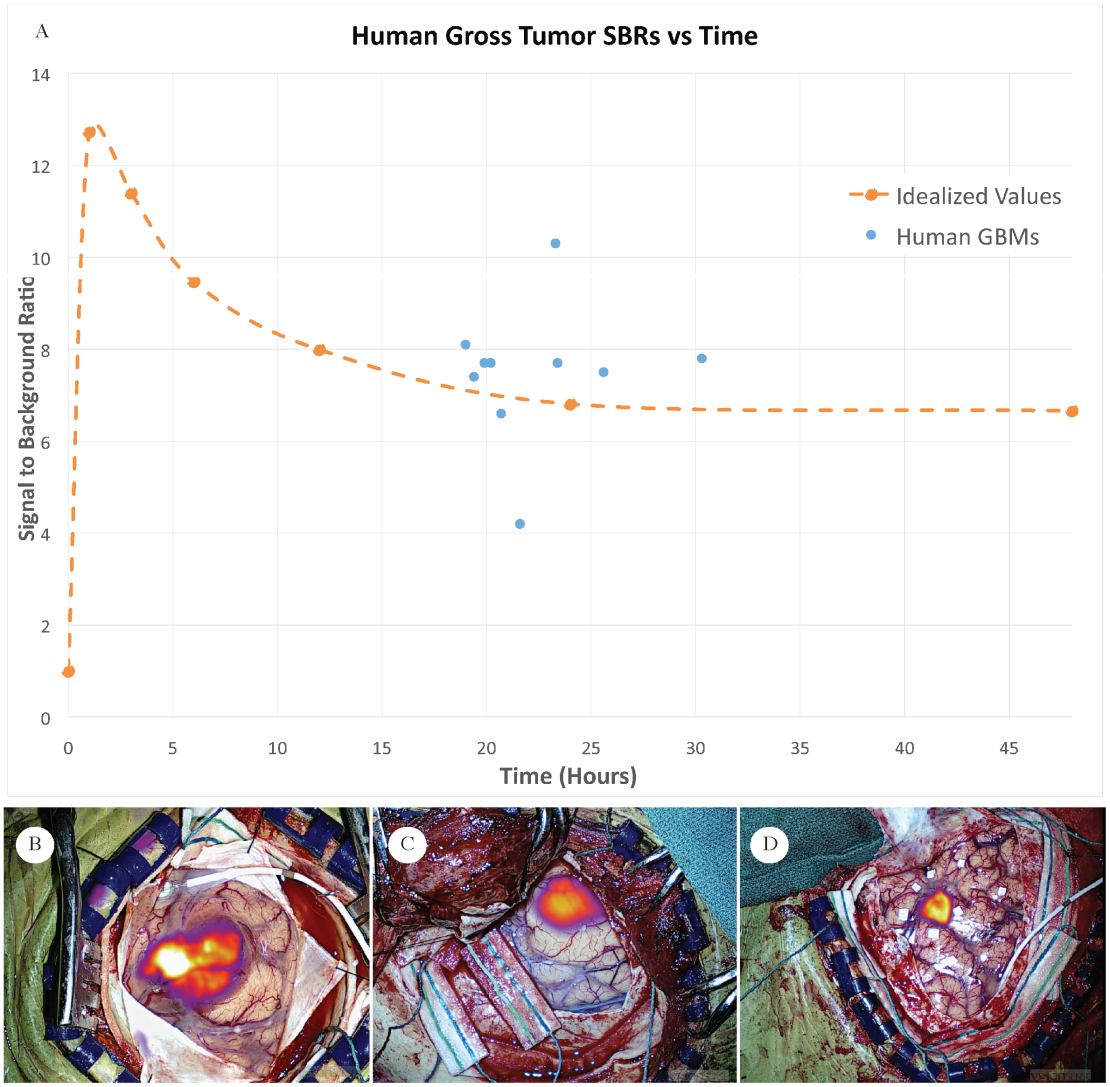
Second Window ICG in humans. A) Signal to background ratios of the human glioblastoma gross tumors plotted (blue dots) versus time of imaging after intravenous ICG injection. The orange line represents the kinetics of SWIG fluorescence obtained from the animal model scaled to humans as described. This is an idealized extrapolation of murine kinetics to the human model by simple multiplication. B, C, D) Subject ID 61, 50, 43 respectively. VisionSense Iridium Near Infrared color-mapped in real time to visible light color view of human brain and tumor after dural exposure.

**Fig 3:**
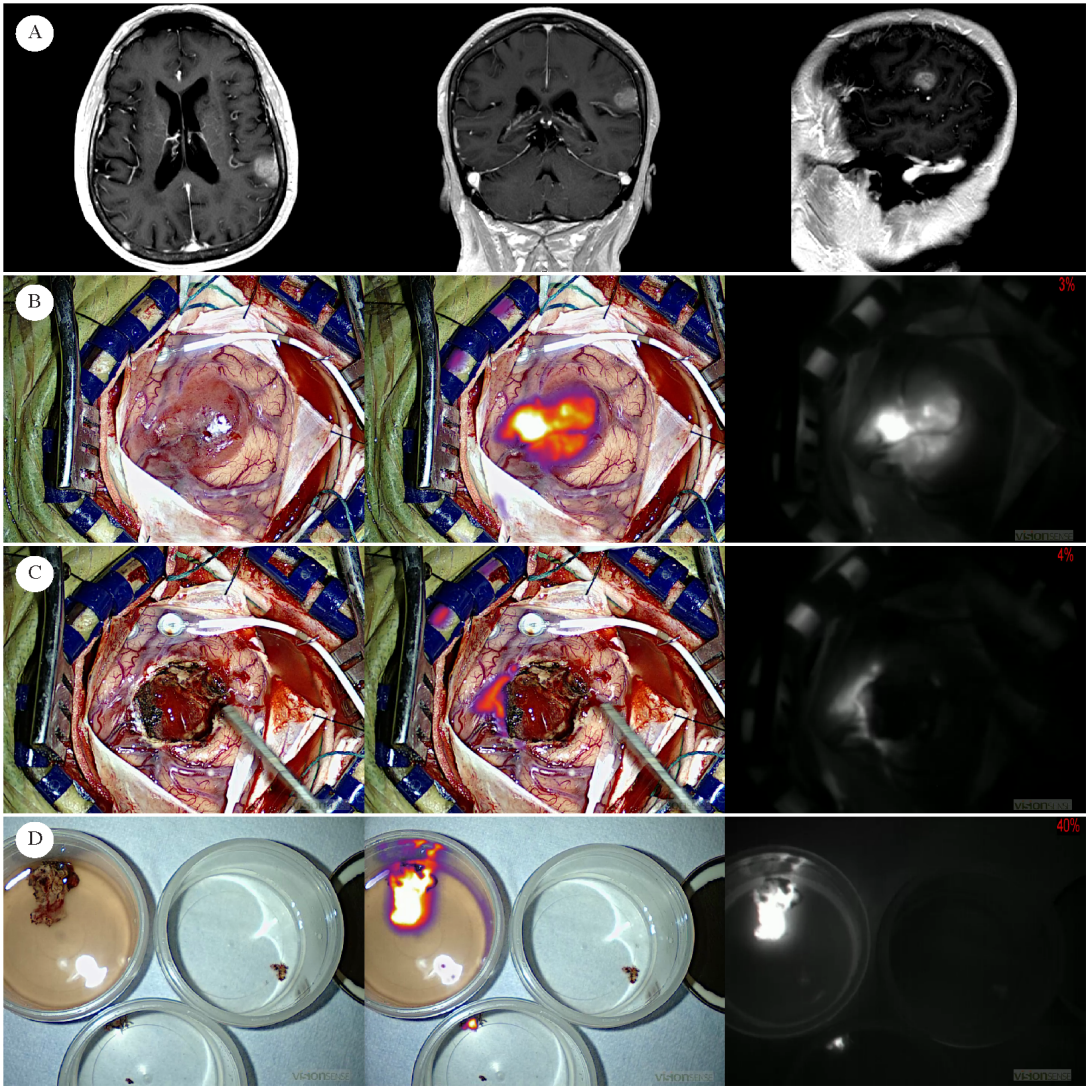
Human left parietal lobe glioblastoma. Row A) Preoperative MRI of newly diagnosed GBM in 63-year-old female (subject ID 61) Subject was injected 20.2 hours prior to visualization of tumor with near infrared–“Second Window ICG” technique (SBR = 7.7). For all images, the left most column presents visible light only. The middle column presents fused near infrared superimposed on visible light, and the right most column presents the NIR view alone. Each of these views is available to the surgeon in real time intraoperatively. Row B) After dural opening, the tumor is grossly visible with visible light alone. NIR signal is very strong. Row C) After conventional resection using white light microscopy only, surgical margin is visualized. Residual signal is biopsied for sensitivity/specificity analysis. Row D) Because resection is performed piecemeal, pieces of tumor are placed in specimen cups in saline. Images of gross tumor (top left), and two biopsy specimen (top right and bottom) ex vivo. After signal analysis, each of these specimen were sent to an independent pathologist for histopathological analysis and confirmed as glioblastoma.

**Fig 4:**
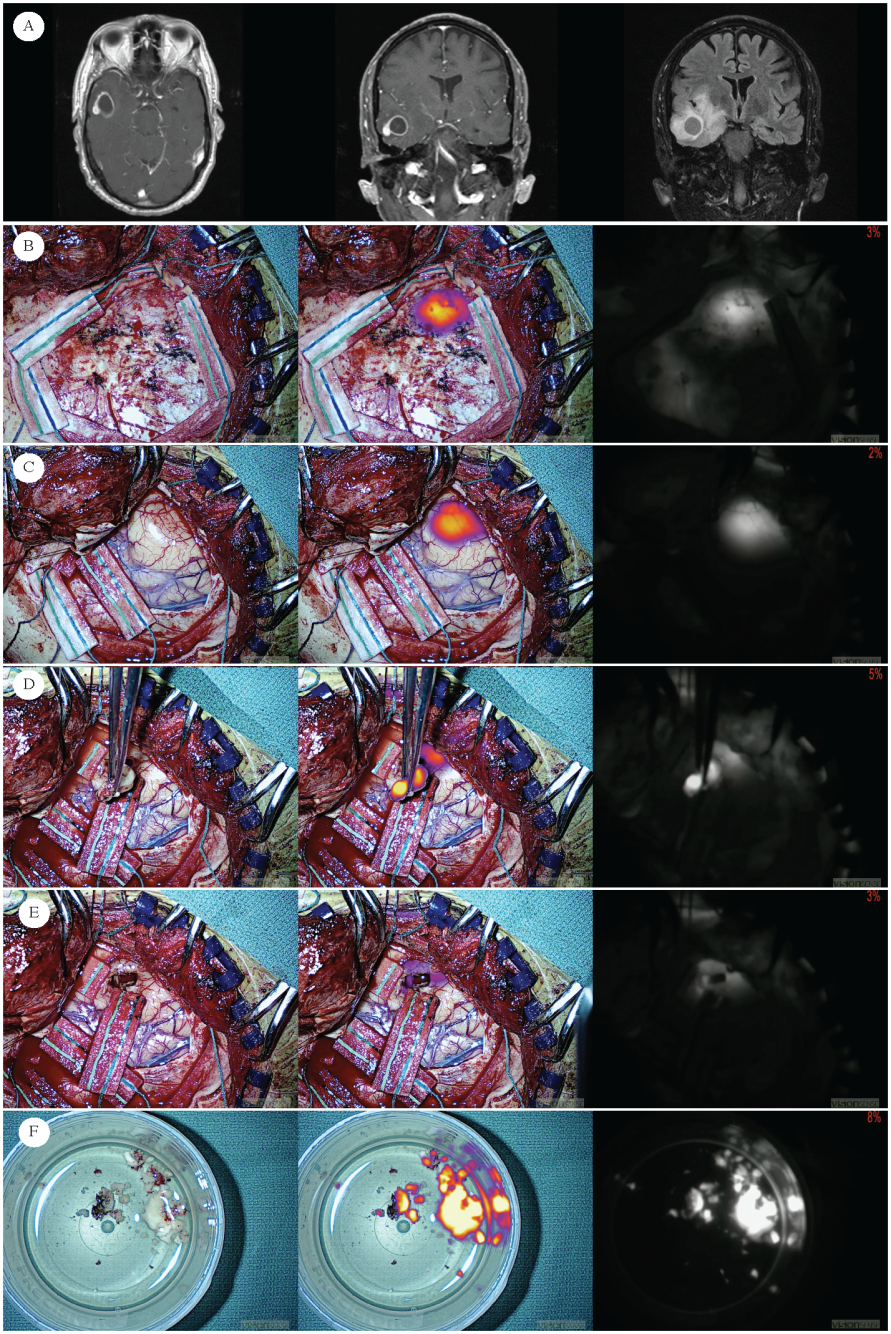
Human right temporal lobe glioblastoma. Row A) Preoperative MRI of newly diagnosed GBM in 65-year-old male (subject ID 50) Subject was injected 19.9 hours prior to visualization of tumor with near infrared–“Second Window ICG” technique (SBR = 7.70). For all images, the left most columns present visible light only. The middle columns present fused near infrared superimposed on visible light, and the right most columns present the NIR view alone. Row B) Before the dura is opened, the tumor cannot be identified with visible light alone. In contrast, because of the longer wavelength of NIR, excitation light and emission can be detected by the NIR camera even through the dura, thus allowing for early localization. Row C) Similarly, after dural opening, the tumor cannot be identified with visible light alone, but with NIR, tumor can be identified even prior to corticectomy. Row D) Gross specimen can be visualized after conventional resection. Row E) After conventional resection using white light only, surgical margin is visualized. Residual signal is biopsied for sensitivity/specificity analysis. Row F). Images of gross tumor ex vivo. Because resection is performed piecemeal, pieces of tumor are placed in specimen cups in saline.

This prospective cohort study, approved by the University of Pennsylvania Institutional Review Board (clintrals.gov, NCT02280954), is open to all patients over the age of 18 undergoing craniotomy for brain tumor (Fig 2). Each patient enrolled provided written informed consent. Based on previous pre-clinical data, all patients were given an infusion of 5.0mg/kg ICG the day before surgery. Because surgery times varied and injections could not be scheduled exactly at 24 hours prior to surgery, a natural experiment was performed in which variable amounts of time transpired before imaging of the tumor in vivo. Fluorescent signal was measured from the gross tumor in vivo in all cases. Specific time points for NIR imaging included before dural opening (dura view), after dural opening (cortex view), upon tumor exposure (tumor view), upon completion of resection (margin view), and ex vivo image analysis of tumor specimens on the back table (ex vivo view) (Figs 3 and 4). As is standard custom in neurosurgery, surgical resection was performed via internal debulking in a piecemeal fashion. Upon completion of the resection, biopsies of residual fluorescent tissue in the surgical margin were taken only if deemed safe by the senior surgeon. The specimens were then immediately analyzed for fluorescence ex vivo on the back table. Specimens were coded as compatible with tumor based on visible light alone by the surgeon in binary (yes/no format) at the time of biopsy acquisition. Specimens were immediately sent to an independent pathologist for histopathological analysis.

#### NIR imaging and measurement

In the operating room, the VisionSense Iridium (Visionsense, Philadelphia PA) camera system was used for in vivo imaging. This system is an open field imaging system designed for use specifically in the operating room. All room lights are turned off, and all window shades are closed in order to minimize stray ambient light. The system is sterilely draped and hung over the operating field to capture data. The VisionSense Iridium measures fluorescence with a narrow filter band that only captures emission between 825 and 850nm. The system overlays NIR signal onto a high quality visible light view as a heat map (Figs 2B–2D, 3 and 4).

SBRs were calculated upon identification and exposure of the gross tumor specimen using VisionSense Player software. Of note, there was a difference in how ROIs were placed in humans versus the mice. In the mice, the ROI was placed around the spot with the brightest signal intensity with the skull closed, which corresponded to the injection site of the tumor. The background chosen was adjacent tissue near the ear or nape of neck. In the humans, ROIs were placed directly over the visible tumor with an exposed brain. Because the brain was exposed, adjacent brain parenchyma could be chosen for the background.

## Statistical analysis

All statistical analyses were performed using Stata 14.0 (StataCorp LP, College Station, TX). Wilcoxon rank-sum (Mann-Whitney) tests were performed to test for differences in fluorescence emitted by the tumors between the two dosing groups at each imaging time point in the mice. Kruskal-Wallis tests, and Mann-Whitney tests, and t-tests were run to determine if there were any significant differences in SBRs among the various time points within each dosing group. A linear regression was performed to determine if there was any correlation between signal strength and time for gross tumor specimen.

## Results

### ICG localizes to U251 intracranial xenografts in mice

U251 tumors were successfully grafted into 18 out of 20 mice, and all mice survived the procedure. Three mice were excluded due to imperfect ICG tail vein injections. Using the IVIS and VisionSense, the presence of tumors was confirmed using both bioluminescent imaging, after intraperitoneal injection of d-luciferin, and fluorescence imaging, after intravenous injection of ICG (Fig 1). Upon examination of frozen sections of the mouse brains with fluorescent microscope, a strong NIR signal was observed from sections containing tumor, but not sections without tumor (Fig 1D). The presence of tumor was also confirmed upon histological examination (Fig 1C).

### Quantification of fluorescence intensity in mice

For the 5.0 mg/kg group (n = 10), the peak TRE came at 1 hour post injection, with an intensity average of 3.37 x 10^9^ [p/s] / [μW/cm^2^] ± 9.9 x 10^8^. The peak ARE also occurred at 1 hour post infusion with an average value of 8.12 x 10^8^ [p/s/cm^2^/sr] / [μW/cm^2^] ± 2.5 x 10^8^. The peak signal to background ratio, measured by comparing the ARE of the tumor to the ARE of adjacent tissue occurred during this initial reading (SBR = 5.07 ± 0.79). The SBR remained high (>2.5) until after 48 hours post injection (Fig 1).

For the 2.5mg/kg group (n = 7), a similar trend was observed. The peak SBR measured via TRE occurred just one hour following injection (8.12x10^8^ [p/s] / [μW/cm^2^] ± 3.3 x10^8^). The peak intensity measured by ARE was also 1 hour following injection (2.25x10^8^ [p/s/cm^2^/sr] / [μW/cm^2^] ± 6.89 x 10^7^). During this time point, the corresponding SBR was 3.62 ± 0.34. The SBR remained above 2.0 through 48 hours. Even with this lower dose, the tumor remained fluorescent for 48 hours following the injection. (Fig 1)

### Comparison of fluorescence intensity between the two dosages

The absolute fluorescent signal intensity improves with higher doses of ICG administration. There was a significant difference in the total radiant efficiency (TRE) between the 5.0 mg/kg group and 2.5mg/kg group at 3 hours (p = .0593), 6 hours (p = .0251) and 12 hours (p = .0339). The 5.0mg/kg group demonstrated 4.15 times greater overall signal in TRE at their peak. Similarly, there was a statistically significant difference at every time point in average radiant efficiency (ARE) between the 5.0mg/kg versus 2.5mg/kg group at 1 hour (p = .0451), 3 hours (p = .0339), 6 hours (p = .0339), 12 hours (p = 0.0516), 24 hours (p = .0388), 48 hours (p = .0447). The average ARE signal was 4.29 times greater at peak signal.

In addition, the average of the SBR at all time points in the 5.0 mg/kg was 3.5 vs. 2.8 in the 2.5mg/kg group, which approached statistical significance (t-test P = 0.0624). However, because of small numbers at each time point, a statistically significant difference between the tumor signal to background ratio of the two doses at each specific time point could not be confirmed. Nevertheless, there appears to be a slightly higher absolute and ratiometric difference in tumor signal at the higher 5.0mg/kg dose versus the 2.5 mg/kg dose (Fig 1).

### Dose and time kinetics for intracranial Second Window ICG

The pattern of NIR signal was similar for both doses—peak fluorescence occurred at one hour, followed by a drop, and then a relatively stable plateau from six hours up to 48 hours later. After 48 hours the NIR signal dropped precipitously (Fig 1). Data analysis with other imaging systems and software platforms yielded the same results. In the 5.0 mg/kg group, significant differences were found between the SBR at 1 hour and every time point (Kruskal-Wallis; p<0.05). In the 2.5 mg/kg group, there were significant differences in the SBR captured at 1 hour with the 24 and 48-hour time points (Kruskal-Wallis; p<0.05). There were no statistical differences between the 6-hour versus the 12, 24 or 48-hour time points with either the high or low dose (Kruskal-Wallis; p>0.05), confirming the concept of a plateau period–“Second Window of ICG.”

### Clinical utility of timing parameters

A Mann-Whitney test was performed to see if there was any difference between signal strengths at the early time points (1,3,6 hours) versus the delayed time points (24,48 hours). The rationale for grouping the one, three, and six-hour time points together to measure them against the 24 and 48-hour time points grouped together is based on clinical utility. It is easier to inject patients on the morning of the surgery and then to proceed with the surgery. It does require more coordination to inject the patients the day before surgery (24–48 hours earlier).

This test showed that there is a statistically significant difference in SBR at the “day of” time point compared to the “day prior” time point. The mean SBR at early time points was 3.94 versus 2.32 at the later time point (Mann-Whitney; p<0.05). This statistically significant difference in SBR was present even if stratified by high and low dose (Mann-Whitney; p<0.05 for all comparisons). Although there is a difference between early and late time points, analysis also revealed that there is no difference in SBR between after 6 hours) (P>.05). These time points, corresponding to the plateau period, thus may represent the best time to visualize tumors with Second Window ICG as there is less variability in signal strength during this time.

### Human clinical study

#### Overview

A total of 10 glioblastoma patients enrolled in this human clinical study were selected for this analysis. There were 5 males and 5 females. The mean age was 61.6 years old (range: 30–81). The range of time elapsed from ICG infusion to visualization of tumor in the operating from was 19.0 hours to 30.3 hours. On average, the time of imaging was 22.3 ± 1.74 hours (Table 1) (Figs 2, 3 and 4).

**Table 1:** Human clinical trial subject study characteristics.

ID	Age	Sex	Tumor Location	Total Dose (mg)	Time from Infusion to Visualization (hours)	Number of Biopsies Analyzed	Months Since Last Intervention	Maximum Tumor Diameter on Pre-Op MRI (mm)	Signal to Background Ratio
19	46	F	Left Frontal	336.5	23.4	3		19.1	7.7
34	59	F	Right Occipital	335.5	19.0	2		38.6	8.1
43	81	M	Right Temporal	402.5	19.4	2		32.6	7.4
46	71	M	Left Parietal	447.0	30.3	4		41.7	7.8
50	65	M	Right Temporal	431.0	19.9	2	17	29.4	7.7
54	56	F	Left Temporal	313.0	20.7	4			6.6
58	30	M	Left Cerebellar	375.0	21.6	1		25.9	4.2
61	63	F	Left Parietal	424.0	20.2	3		23.7	7.7
63	69	F	Left Frontal	367.5	25.6	2	8	45.1	7.5

Using the VisionSense system for image analysis in the operating room, the average signal to background ratio for the main tumor specimen was 7.50 ± 0.74 (Figs 2, 3 and 4). Regression analysis did not identify time from injection to be associated with the SBR of the glioblastoma in humans within the 19 to 30 hour timeframe (R^2^ = 0.019). This lack of time dependence in this plateau period of 19–30 hours suggests that there is a useful window period in which to image ICG accumulation with the “Second Window of ICG” technique.

Diagnostic tests were performed in order to determine the sensitivity, specificity, positive and negative predictive values of this technique. In addition to each patient’s gross tumor, a total of 15 specimens were biopsied from the 10 patients included in this analysis (examples: Figs 3 and 4). The median SBR of all specimen in this analysis was 5.70 (IQR = 2.7–8.2). At the time of biopsy, the specimen was immediately coded as positive for tumor or negative for tumor using visible light only by the senior surgeon. The NIR imaging device was then immediately used to image the specimen, which was accordingly coded as positive or negative for fluorescence. Coding from both the main tumors and surgical margin biopsy specimen were histopathologicaly correlated using an independent pathologist’s reading as the gold standard. Table 2 displays the diagnostic characteristics from the patients included in this analysis.

**Table 2:** Second Window ICG diagnostic characteristics.

Visible Light vs. Final Pathology	
True Positives: 13	False Positives: 0
False Negatives: 4	True Negatives: 8
Sensitivity: 76.5% (95% CI: 50.1%-93.2)	Specificity: 100.0% (95% CI: 63.1% - 100%)
Positive Predictive Value: 100.0% (95%CI– 75.3% - 100%)	Negative Predictive Value: 66.7% (95% CI: 34.9% - 90.1%)
NIR Light vs. Final Pathology	
True Positives: 18	False Positives: 3
False Negatives:3	True Negatives: 1
Sensitivity: 85.7% (95% CI: 63.7% - 97%)	Specificity: 25.0% (95% CI: 0.63% - 80.6%)
Positive Predictive Value: 85.7% (95% CI:63.7% - 97%)	Negative Predictive Value: 25.0% (95% CI: 0.63% - 80.6%)

The results from this analysis corroborate previously published findings on the value of this technique as having a higher sensitivity than the naked eye alone, but this comes at the expense of specificity (17). With the limited number of patients and specimens, however, the confidence intervals for the test characteristics are extremely wide. For example, the point estimate specificity of NIR for tumor is 25%, but the confidence interval spans from just under 1 percent to 80 percent.

#### Results in light of murine model kinetics

A comparison of the human glioma NIR signal versus the murine NIR signal demonstrates stark difference in SBR. In contrast to the human SBR at 24 hours of 7.50 ± 0.74, the average SBR at the 24-hour time point in mice was 2.55 ± 0.68. This difference in SBR may be related to the fact that in the human study, we were able to obtain ROI in normal brain parenchyma (Figs 3 and 4), but this was not possible in mice. In the mice, the imaging was performed through an intact skull, and thus signal intensity is likely compromised.

In order to compare the human findings to the murine time experiment, an idealized SBR vs. time trend line was plotted to illustrate where the SBR of the human specimen lies in relation to the mouse model study. This idealized plot was obtained by multiplying the murine time points by the 24-hour time point SBR ratio (7.28/2.55) **(**Fig 2A). Although murine and human pharmacokinetics are not interchangeable, we hypothesized that the plateau period of ICG accumulation and nonspecific retention within tumors was generally comparable. Thus, the extrapolated trend line in Fig 2 is intended to provide graphic appreciation of where the human patients’ tumors are being imaged.

## Discussion

Complete resection of glioblastoma without neurologic deficit remains the primary goal of every neurosurgeon, and the use of intraoperative visible light fluorescence imaging with 5-ALA has been shown to result in greater extent of resection, and thus greater progression free survival [5, 19]. Despite these advantages, 5-ALA is not FDA-approved. Indocyanine green, however, is a readily available, FDA-approved NIR fluorescent drug which has promise as a tumor optical contrast agent [11–16]. Although the Second Window ICG technique has been studied in a rodent model of lung cancer, we hypothesized that Second Window ICG could also provide optical contrast of brain tumors. In this study we demonstrate that this technique indeed provides strong NIR optical contrast in both the rodent model and the human subjects. We subsequently explored the optimum timing and dosing for the accumulation of ICG in these tumors using this technique.

### Optimal time of imaging—How long is the ‘Second Window’ open?

For purposes of clinical tumor identification, we distinguish two periods in the kinetics of Second Window ICG for fluorescent-guided surgery of brain tumors. The first early peak contains the signal’s maximum absolute intensity. The peak signals occurred one hour after ICG administration. This peak can be appreciated for up to 6 hours before the signal declines and plateaus. The SBR at these early time points was over a full point higher than the later time points. This effect was not compromised when stratifying the data by dose. Although SBR accounts for background, the observed increase in SBR at these early time points may be due to the amount of large concentration and volume of ICG initially flooding tissue via vasculature. Although ICG accumulation appears to be very strong during this period, we have not chosen to visualize NIR signal accumulation in tumors during this period, as we believe the differences in signal over time will be quite large, and thus minor differences in time from infusion will result in large changes in signal, making comparison across patients challenging.

In contrast, we are interested in the plateau phase of “Second Window of ICG” accumulation within intracranial tumors. We hypothesize that this period begins at 6 hours and lasts for at least 48 hours after intravenous injection of ICG. Statistical analysis showed no difference in signal strength at any of the time points within this plateau for both mice and humans. Thus, it is possible that imaging during this period allows for uniform signal characteristics even across subjects. This was confirmed when analyzing the human participants. There was no significant difference seen in NIR signal in the time range of imaging between 19 and 30 hours.

From a clinical standpoint, the authors hypothesize that Second Window ICG can be used to visualize intracranial tumors with a greater absolute signal following a same-day ICG administration albeit at the expense of a rapidly changing signal during this dynamic period. In contrast, Second Window ICG can also be used to visualize tumors with perhaps more consistent tumor contrast at delayed time points such as 24 hours after IV administration.

### Optimal Dose for Second Window ICG

In this study the higher dose of 5.0 mg/kg versus 2.5 mg/kg appears to result in a modest improvement in absolute NIR signal. The absolute signal intensity, as measured by radiant efficiency, was enhanced in the higher dose group compared to the lower dose group. In addition, the average SBR from all time points was almost a full point higher in the 5.0 mg/kg dose group compared to the lower dose group. Although this observation approached statistical significance, we were unable to confirm a statistically significant (p<0.05) difference between the two doses at individual time points. We could not test this hypothesis in our human study as we only administered 5.0 mg/kg dosing in our study. Nevertheless, we believe it may be possible to visualize tumors via Second Window ICG with 2.5 mg/kg rather than 5.0 mg/kg. This may result in some savings with respect to dye costs. Because of our current success with the 5.0 mg/kg dose, we currently do not have plans to change our doses in human patients. Additionally, although the quantum efficiency of ICG is very low for a fluorophore, we do believe that it is hypothetically possible to witness quenching in some brain tumors. This phenomenon may be a reason to lower the ICG dose in some tumors, but this possibility remains to be researched in future studies.

### Comparison to prior studies with ICG

The dosing and timing of dye administration for Second Window ICG was optimized in preclinical rodent studies utilizing esophageal and lung cancer cell lines in subcutaneous flank tumor models. In these studies, the optimal tumor versus normal tissue contrast came 24 hours after intravenous administration of ICG doses greater than or equal to 5.0 mg/kg [9, 11, 13, 14, 17, 20]. In the flank tumor model, the NIR signal in the tumor is competing with other places that ICG can accumulate, (e.g. bowel, liver, lymph nodes). Indeed, ICG is metabolized by the liver and thus optimal signal take into account the strong liver background. Thus, when visualizing tumors in the flank, more time is needed for normal tissue to clear the ICG before adequate contrast between the tumor and normal tissue can be observed. Intracranial tumors, in contrast, do not have significant ICG accumulation in any organ with any significant spatial proximity to the brain. As such, tumor signal may be more easily identified at earlier time points. Similarly, because of the lack of any significant ICG accumulation in adjacent organs near the brain, the lower dose of 2.5 mg/kg may be sufficient to provide strong NIR signal in human glioblastoma patients.

### NIR signal intensity

We observed a three times stronger NIR signal in human glioblastoma subjects as compared to the rodent tumor model. Although murine pharmacokinetics are not always generalizable to humans, this difference can additionally be explained by several factors. First, NIR imaging was perfo**r**med through an intact skull in the mice which results in a weaker NIR signal. In contrast, in human subjects, the NIR signal was captured after the skull had been removed via craniotomy. Thus, the signal and background were both obtained directly from tumor and adjacent brain parenchyma. In the operating room, when we tried to visualize NIR signal through the skull, this was not possible. Another consideration is that the imaging system was different for the mice and the humans as the closed field IVIS system cannot be used for human patients. We did, however, have some mice where we imaged their tumors with both the IVIS and the Visionsense, and we did not see any significant differences between the signal intensities. Hence, we do not believe that this is a significant source of variability. A third possibility, is that NIR signals are higher in human glioblastoma because the tumors are typically much larger, and thus can accumulate more ICG.

### Analysis of human data

There was a moderate increase in sensitivity to detect tumor using Second Window ICG compared to traditional surgical techniques. However, this increase in sensitivity comes at the expense of sensitivity. Furthermore, the confidence intervals for these test characteristics are extremely wide.

The average SBR of the gross tumor in all subjects was very high (mean = 7.5), and the signal could be additionally appreciated both through the dura and through limited cortex (Fig 4). These results are in agreement with prior findings in a study on 18 patients using this technique for glioma resection as published by Lee et al., 2016.

### Limitations of Second Window ICG

Although this study provides evidence that Second Window ICG can be used for intracranial tumor visualization, there are several limitations to this study. Only one type of tumor histology was studied in the animal model. It is possible that other cell lines with differing genomic characteristics and growth patterns respond differently to Second Window ICG. Also, different camera systems were used in the mice and the humans, and pharmacokinetics between the species is not completely commutable. Thus truly direct comparisons can only be inferred. Additionally, although there has been extensive research on the enhanced permeability and retention effect, why ICG accumulates in intracranial tumors is not completely understood [10, 21]. ICG is not receptor-bound, so its location in the tumor environment is not precisely known. Indeed, our own correlation with pathology, as published by Lee et al., 2016 suggests that the sensitivity is high but the specificity is low [17]. Despite these limitations, we believe Second Window ICG has an exciting role to play in fluorescent-guided surgery for brain tumors.

### Benefit of Second Window ICG

The 5-ALA Glioma Study group in Europe published a randomized clinical trial by Stummer et al [19]. Oral administration of the prodrug 5-ALA, which is converted to protoporyphyrin IX was used to visualize tumor cells in vivo at the time of surgery. Patients that received 5-ALA were more than twice as likely to have gross total resection and had dramatic improvement in progression free survival. Additionally, two single center retrospective studies by Sanai et al and LaCroix et al demonstrated improvement in survival with greater extent of resection, and a post hoc analysis of three prospective Radiation Therapy Oncology Group (RTOG) trials demonstrate inferior results with biopsy and pharmaceutical treatment only [22–25]. For these reasons, improvements in safe resection of glioblastoma are paramount. This study provides foundational work to exploit the Second Window ICG in glioblastoma resection.

## Conclusion

The Second Window ICG technique for near-infrared, optical tumor contrast is a novel technique that can be readily performed in the neurosurgical operating room theater. This technique exploits the enhanced permeability and retention effect for delivering ICG to a variety of tumor types, and in this study, its application was explored in both an orthotopic glioblastoma model in both mice, and in humans with glioblastoma. Because of the unique characteristics of the blood-brain barrier and lack of any significant ICG accumulation in nearby head and neck structures, we hypothesize that the “Second Window” for intracranial tumor visualization is open as early as a few hours after intravenous ICG administration, and the signal can be appreciated during a relatively long and stable period of time during a plateau phase. We believe this technique can have useful application for early, accurate identification of glioblastoma in humans during surgery.

## Supporting information

S1 Table Raw fluorescence and descriptive data. Page 1: Raw data from enrolled human subjects, including demographic information and diagnostic tests. Page 2: Raw data from murine experiments including average and total radiant efficiency, signal to background ratios and summarized averages.
(XLSX)
